# Prevalence of ‘*Candidatus* Rickettsia vini’ in *Ixodes arboricola* ticks in the North of Spain, 2011–2013

**DOI:** 10.1186/s13071-015-0724-6

**Published:** 2015-02-19

**Authors:** Ana M Palomar, Aránzazu Portillo, Ariñe Crespo, Sonia Santibáñez, David Mazuelas, José A Oteo

**Affiliations:** Departamento de Enfermedades Infecciosas, Center of Rickettsioses and Arthropod-Borne Diseases, Hospital San Pedro-CIBIR, 98-7ª N.E, 26006 Logroño, La Rioja Spain; Aranzadi Society of Sciences, San Sebastián, Guipúzcoa Spain; Abies, Environment Resources Inc, Logroño, La Rioja Spain

**Keywords:** *Candidatus* Rickettsia vini, *Ixodes arboricola*, Bird, Spain

## Abstract

**Background:**

The prevalence of *Rickettsia* spp. in *Ixodes arboricola* ticks collected from birds in two locations in the North of Spain from 2011 to 2013 was studied.

**Findings:**

The detection of the bacteria in 54 DNA extracts of *I. arboricola* was performed by PCR targeting an *ompA* fragment gene. The 94.4% of the samples yielded positive results and the nucleotide sequences were homologous (100% identity) to ‘*Candidatus* Rickettsia vini’.

**Conclusion:**

The high rate of infection suggests that ‘*Ca.* R. vini’ is a common endosymbiont of *I. arboricola*.

## Background

The worldwide-distributed *Rickettsia* genus (family Rickettsiaceae; order Rickettsiales; class α-Proteobacteria) includes Gram-negative, small, obligate intracellular, non-motile, pleomorphic, coccobacilli bacteria transmitted by arthropods. Ticks are among the main vectors of these agents and can also act as reservoirs of them [1]. Up to date, about 30 *Rickettsia* species have been described and this number is increasing [1,2]. In addition, a large number of rickettsiae have been genetically characterized but remain uncultured due to the fastidious nature of these bacteria. For them, and according to taxonomic criteria, ‘*Candidatus’* statuses are proposed [3]. This is the case of ‘*Candidatus* Rickettsia vini’ that was discovered in immature *Ixodes arboricola* and *Ixodes ricinus* collected from birds in 2009 in the North of Spain [4,5]. Subsequently, this bacterium was detected in two pools of *I. arboricola* removed from birds in Turkey in 2012 [6]. As it occurs with other *Rickettsia* spp., the pathogenic role of ‘*Ca.* R. vini’ remains unknown. Nevertheless, all *Rickettsia* species identified in arthropods should be considered potentially pathogenic for humans. Most human pathogenic *Rickettsia* spp. have been initially identified in arthropods before being detected in human samples [1]. Due to the scarce information about ‘*Ca.* R. vini’, this study aimed to investigate its prevalence in the host *I. arboricola* tick.

## Findings

A total of 56 ticks (17 larvae, 36 nymphs and three females) morphologically classified as *I. arboricola* through taxonomic keys [7] were selected from the tick collection of the Center of Rickettsiosis and Arthropod-Borne Diseases in Logroño, Spain. Ticks were collected from birds in the North of Spain from 2011 to 2013. Two of them were collected from a European Greenfinch (*Cholis cholis*) in Ribafrecha (La Rioja, Spain). These arthropods arrived alive at the laboratory and were frozen at −80°C. The remaining 54 *I. arboricola* specimens were collected from seven Eurasian Blue Tit (*Cyanistes caeruleus*), four Great Tit (*Parus major*) and two Marsh Tit (*Poecile palustris*) in Eulate (Navarra, Spain), and preserved in 70% ethanol. The number and stage of the selected ticks, as well as the host bird species is shown in Table 1.

**Table 1:** **Prevalence of ‘** ***Candidatus*** **Rickettsia vini’ in** ***Ixodes arboricola*** **in the North of Spain, 2011–2013**

**Species of birds (No. of specimens)**	**Location**	**Ticks No./stage**	**Prevalence of ‘** ***Ca.*** **R. vini’ (%)**		
			**L**	**N**	**F**
*Cholis cholis* (1)	Ribafrecha (42°21N; 2°24′W)	2L	100		
*Cyanistes caeruleus* (7)	Eulate (42°46′N; 2°12′W)	3L; 8N*; 3F	100	86	100
*Parus major* (4)	Eulate	10L*;27N	100	93	
*Poecile palustris* (2)	Eulate	2L;1N	100	100	

*One specimen was not tested for the presence of spotted fever group *Rickettsia.*

No.: Number; ‘*Ca*. R. vini’: ‘*Candidatus* Rickettsia vini’; L: Larva; N: Nymph; F: Adult female.

DNA was individually extracted from the tick samples using two incubations of 20 minutes each with ammonium hydroxide (1 mL of 25% ammonia and 19 mL of MQ water) at 100 and 90°C, respectively. A single PCR targeting the 16S mitochondrial genome of ticks (16S rRNA) was performed for all the samples to validate the DNA extraction method [8]. All 16S rRNA PCR products were sequenced in one sense to confirm the morphologic classification of ticks through genetic approaches. In these cases, DNA extracts were further tested for spotted fever group (SFG) *Rickettsia* using a semi-nested PCR for *ompA* [9,10]. This PCR assay was selected according to the previously demonstrated sensitivity and usefulness for species identification [11]. Two negative controls, one of them containing water instead of template DNA and the other with template DNA but without primers, as well as a positive control of *Rickettsia slovaca* strain S14ab DNA (obtained from a culture of a *Dermacentor marginatus* tick in Vero cells) were included in all PCR assays. All *ompA* amplicons were sequenced in both senses, and nucleotide sequences were compared with those available in NCBI database using BLAST (http://blast.ncbi.nlm.nih.gov/Blast.cgi).

A total of 54 out of 56 *I. arboricola* (all but one nymph and one larva) tested positive for the 16S rRNA PCR. The analysis of these 54 nucleotide sequences (358–385 bp) revealed 100% identity with the 16S rRNA gene from *I. arboricola* [GenBank: JF791813 and JF91812 for 35 and 19 cases, respectively]. The screening of SFG *Rickettsia* was performed in all but the two samples that did not amplify the 16S RNA gene. Based on rickettsial *ompA* PCR results, SFG *Rickettsia* was found in 94.4% specimens (51/54). Three nymphs were found to be negative by *ompA* PCR. The analysis of *ompA* nucleotide sequences (590 bp) showed 100% identity with *ompA* of ‘*Ca.* R. vini’ [GenBank: JF758828]. The prevalences of ‘*Ca.* R. vini’ for larvae and female adults were 100% and reached 92% for nymphs. Details for each bird species and stage of the *I. arboricola* ticks are shown in Table 1.

In this study we report the detection of ‘*Ca.* R. vini’ in 94.4% *I. arboricola* collected from birds in two locations of the North of Spain from 2011 to 2013.

*I. arboricola* is a nidiculous ectoparasite of bats and birds that principally infests bird species that nest in cavities. Among them, Blue and Great Tits are the main hosts [12]. In our study, *C. caeruleus*, *P. major* and *P. palustris* (bird species that nest in tree holes) were found parasitized by *I. arboricola* in the Navarra Province. Moreover, a unique specimen of *C. cholis* was found infested by two *I. arboricola* ticks in La Rioja Province. This finding was unexpected since this bird species nests in trees but not in cavities. The host specificity of *I. arboricola* seems to be limited by host ecology rather than host specialization [12]. In this way, an accidental intrusion of this bird specimen on the nest of a cavity-nesting bird species could explain its infestation, although *C. cholis* does not seem to be a common host.

Up to date, only three studies report the presence of *Rickettsia* spp. in *I. arboricola* specimens [4,6,13]. Spitalská and colleagues detected rickettsia infection in *I. arboricola* removed from birds in the Czech Republic from 2003 and 2005, with prevalence of 44% in larvae and 24.5% in nymphs [13]. In this case, *Rickettsia* spp. cannot be identified since only partial sequences of a highly conserved fragment of the *gltA* gene are available. Our group found a prevalence of *Rickettsia* spp. in the 100% of 25 immature *I. arboricola* specimens collected from a Blue Tit and a Great Tit in 2009 in an area located in La Rioja (42°14′N, 2°54′W) [4]. This *Rickettsia* was genetically characterized and named ‘*Ca.* R. vini’ [5]. In the same study, two *I. ricinus* specimens infected by ‘*Ca.* R. vini’ were also detected (0.01%). Both were removed from *Erithacus rubecula* specimens. It is known that this bird species occasionally nests in natural cavities, thus enabling that *I. ricinus* and *I. arboricola* share host bird. Therefore, and joined to the low prevalence of ‘*Ca.* R. vini’ in *I. ricinus* and the fact that they were engorged and collected at the same time and place as *I. arboricola* infected by ‘*Ca.* R. vini’, the finding of the bacterium in *I. ricinus* does not involve this tick species as competent vector or reservoir, although the data should be taken into account. In the third study mentioned above, Keskin and colleagues tested two pools of *I. arboricola* (two nymphs and five larvae) collected from birds in Turkey in 2012, detecting ‘*Ca.* R. vini’ in both of them (100%) [6]. Up to this study, ‘*Ca.* R. vini’ had been studied in a very limited number of samples that had been collected over few bird specimens. Our work provides important information about the prevalence of the bacterium in *I. arboricola* of all stages removed from a higher number of bird specimens (n = 14) and in different periods of time (2011–2013). ‘*Ca.* R. vini’ has been detected in 94.4% ticks, reaching 100% prevalence for all stages except for nymphs (92%).

## Conclusions

According to our results, we can confirm a high infection rate of *I. arboricola* by ‘*Ca.* R. vini’. Our data suggest that ‘*Ca.* R. vini’ is a common endosymbiont of *I. arboricola*. More studies are needed to know the role of *I. arboricola* as vector and reservoir of ‘*Ca.* R. vini’, the role of birds and bats (hosts of *I. arboricola*) as reservoirs or amplifiers of this microorganism, as well as the potential pathogenicity of this ‘*Candidatus’* for humans and animals. Nevertheless, and up to our knowledge, *I. arboricola* ticks do not bite humans.
